# Molecular Endoscopic Ultrasound for Diagnosis of Pancreatic Cancer

**DOI:** 10.3390/cancers3010872

**Published:** 2011-02-24

**Authors:** Barbara Bournet, Adeline Pointreau, Yannick Delpu, Janick Selves, Jerome Torrisani, Louis Buscail, Pierre Cordelier

**Affiliations:** 1 Department of Gastroenterology, University Hospital Center Rangueil, 1 avenue Jean Poulhès, TSA 50032, 31059 Toulouse Cedex 9, France; E-Mail: bournet.b@chu-toulouse.fr; 2 INSERM U1037, University Hospital Center Rangueil, Toulouse, France; E-Mails: apointreau@yahoo.fr (A.P.); yannick.delpu@inserm.fr (Y.D.); selves.j@chu-toulouse.fr (J.S.); jerome.torrisani@inserm.fr (J.T.); Pierre.cordelier@inserm.fr (P.C.)

**Keywords:** endoscopic ultrasound-guided fine needle aspiration-biopsy, pancreatic ductal adenocarcinoma, *KRAS* oncogene, microRNA, chronic pancreatitis

## Abstract

Endoscopic ultrasound-guided fine needle aspiration-biopsy is a safe and effective technique in diagnosing and staging of pancreatic ductal adenocarcinoma. However its predictive negative value does not exceed 50% to 60%. Unfortunately, the majority of pancreatic cancer patients have a metastatic and/or a locally advanced disease (*i.e.*, not eligible for curative resection) which explains the limited access to pancreatic tissue specimens. Endoscopic ultrasound-guided fine needle aspiration-biopsy is the most widely used approach for cytological and histological material sampling in these situations used in up to two thirds of patients with pancreatic cancer. Based on this unique material, we and others developed strategies to improve the differential diagnosis between carcinoma and inflammatory pancreatic lesions by analysis of *KRAS* oncogene mutation, microRNA expression and methylation, as well as mRNA expression using both qRT-PCR and Low Density Array Taqman analysis. Indeed, differentiating pancreatic cancer from pseudotumoral chronic pancreatitis remains very difficult in current clinical practice, and endoscopic ultrasound-guided fine needle aspiration-biopsy analysis proved to be very helpful. In this review, we will compile the clinical and molecular advantages of using endoscopic ultrasound-guided fine needle aspiration-biopsy in managing pancreatic cancer.

## 1. Background

Pancreatic cancer remains one of the most deadly types of tumor. The five year survival rate after diagnosis is less than 3.5% [1,2]. Only 12% to 17% of pancreatic ductal adenocarcinoma (PDAC) patients can be diagnosed at a resectable and possible curative stage. The remaining patients diagnosed with locally advanced and/or metastatic tumors are offered some relief with palliative care. Single-agent gemcitabine, although not dramatically improving survival, has demonstrated a significant clinical benefit and has become the standard chemotherapy for advanced PDAC [3,4]. Recently, FOLFIRINOX therapeutic regimen was found to improve the survival of metastatic patients when compared to gemcitabine alone [5]. However, median survival does not exceed six and nine months for metastatic and locally advanced PDAC, respectively [2-4]. One way to improve PDAC management is to establish a diagnosis at an early curative stage. In the absence of specific risk factors, there is no high-risk patient group to target for possible screening except for members of pancreatic cancer-prone families. In addition, there is no highly sensitive and specific serum marker available to date. However, recent advances in abdominal imaging techniques may favor a more rapid histological diagnosis and also may resolve several problems of differential diagnosis. A problematic clinical condition is the differentiation of PDAC from focal pancreatitis. It is indeed critical to avoid unnecessary resection of benign lesions (such as focal lesions of chronic pancreatitis or autoimmune pancreatitis) or to delay the treatment of PDAC in a subset of patients. However, despite modern imaging techniques, difficulties persist to early diagnose PDAC and to differentiate PDAC from benign diseases such as chronic pancreatitis (CP) especially in its pseudotumoral form [6,7]. Nowadays, endoscopic ultrasound (EUS) takes a significant share of the diagnosis and management of gastro-entero-pancreatic and biliary diseases [8,9]. Recent technical breakthroughs turned EUS into an interventional procedure, thus rendering fine needle aspiration/biopsies (FNAB) possible. In this way, EUS allows to safely guide FNA of solid or cystic peri-digestive or pancreatic lesions for cytopathological analysis. Endoscopic ultrasound-guided fine needle aspiration-biopsy (EUS-FNAB) is a safe and effective technique in diagnosing and staging of PDAC [9-12]. However, its accuracy for the diagnosis of malignancy varies widely with a sensitivity ranging from 65% to 95%, and with a mean accuracy of 85% (negative predictive value ranging from 50% to 70 %). Additionally, EUS-FNAB may be inconclusive in up to 20% of cases [9-14]. Despite the miniaturization of histological samples provided by the FNAB using 22 Gauge needle, immunohistochemistry can be achieved when micro biopsies are collected, fixed and embedded in paraffin. Immunodiagnostic can be useful to differentiate for instance PDAC from endocrine tumors. It is harder to differentiate malignant from inflammatory lesions of exocrine pancreas. In parallel, the improvement of molecular biology techniques including DNA and RNA amplification permits the analysis and the quantification of molecular markers in cytological samples. In addition, EUS-FNAB is the main clinical appliance for cytological and histological material collection from locally advanced PDAC that represents 85% of pancreatic cancer patients. This chapter depicts the widespread potential for the molecular analysis of samples obtained by EUS-FNAB in assessing diagnosis or prognosis of PDAC, as well as translational studies on new markers and epigenetic alterations.

## 2. Molecular Biology on EUS-Fine Needle Aspiration-Biopsy Samples from Pancreas

### 2.1. DNA Extraction and Analysis

Despite using fine needles of 22G, sufficient materials can be obtained for cytology and histology (*i.e.*, microbiopsy). A portion of this material, collected following air or saline flushing of the needle once the core biopsies have been reclaimed for histopathology, can be used for further molecular analysis. In our experience, a mean of 500 nanograms of DNA (range 150 nanograms to 1.5 micrograms) is obtained and DNA amplification is possible in all cases [15]. For comparison, previous studies and protocols conducted on pure pancreatic juice attested for a lack of extraction/amplification in almost 13% of samples [16-18]. Thereafter, purified DNA authorizes PCR followed by Restriction Fragment Length Polymorphism or sequencing. Recently we developed an allelic discrimination assay on material sampled on EUS-FNAB as well as specific Methylation-Specific PCR assay. All these procedures are successful in almost 100% of the cases, in the absence of DNA pre-amplification. This is of prime importance because DNA amplification generates mutations especially when using a low amount of starting material that can eventually bias subsequent analysis.

### 2.2. RNA Extraction

While material collected from pancreatic tumor or inflammatory tissue is less exposed to nuclease digestion as compared to normal pancreatic tissue, the risk of degradation is very high if one wants to analyze high-quality RNA. From a practical point of view, cytological samples should be immediately stored in transport medium (such as RNable) and frozen at −20 °C until use. Despite all these precautions, difficulties persist. Quality and quantity of RNA must be systematically checked with bioanalyzers (for example Biorad Experion analyzer and Agilent Technologies). Our group estimates that RNA samples that are highly degraded (RNA 18S/28S ratio less than 1) and/or in low quantities (lower than 100 picograms per μL) are usually not suitable for further analysis (mainly qPCR). However, even if quantity of RNA is low (but not degraded) RNA amplification kits are now available and permit up to 500 fold amplification with satisfactory reproducibility and reliability. In other terms, the RNA amplification from EUS-FNA material preserves the pattern of gene expression and is not influenced by the origin of the sample.

## 3. *KRAS* Mutation Assay and Diagnosis of Solid Pancreatic Mass

Several genetic alterations are well characterized in PDAC such as codon-12 *KRAS* mutation (75% to 95%) and to a lesser extent *p16*, *DPC4* and *p53* gene mutations [19,20]. Previous studies conducted by our group and others on pure pancreatic juice obtained by ERCP concluded that *KRAS* mutation was found in 60% to 65% of PDAC [16-18]. However, this approach does not improve the diagnosis of PDAC nor the differential diagnosis of PDAC from CP [17,18,21]. Moreover, the addition of *p16* and *DPC4* mutation analysis in pure pancreatic juice does not improve the sensitivity and specificity of *KRAS* mutation detection for the distinction of PDAC from CP [18]. Further studies revealed that the *KRAS* mutation could be detected in cellular materials obtained by EUS-FNAB [22-25]. With the addition of *KRAS* mutation analysis, EUS-FNAB appeared to be highly accurate for the differentiation of benign *versus* malignant pancreatic solid lesions [15,22-27]. Both monocenter and multicenter studies concluded that *KRAS* mutation analysis might be essential to strengthen the diagnosis of pancreatic masses by EUS. This latter point is essential for the differential diagnosis between PDAC and CP in the subgroup of pseudo-tumorous forms. Results of these studies, including our experience, are detailed in Table 1. Performances of *KRAS* mutation assay alone are similar regardless of the technique used. In addition, DNA extraction has been performed on various types of samples such as cellular materials air blushed from the needle biopsy [15,22,23], total core biopsy removed from the needle [24], or microdissected tissue from fixed/embedded biopsies [27]. Performances of *KRAS* mutation assay were also similar regardless of the method of sampling. Finally, the sensitivity and overall accuracy of PDAC diagnosis is improved when combining cytopathology and *KRAS* analysis (Table 1).

However, *KRAS* alone is not sufficient for diagnosis but in case of suspicious cytopathological results, presence of *KRAS* mutation is evocative of malignancy and may justify a second biopsy and a follow up to exclude PDAC. Conversely, because of the high specificity of *KRAS* assay, the presence of wild type *KRAS* at EUS-FNAB, together with CP-like clinical and radiological profile, is highly evocative of benign lesions. Based on the combination of histo/cytopathological and *KRAS* mutation analysis, a medical or surgical conservative treatment is pertinent for patients with pancreatic solid mass suggestive of pseudo-tumorous chronic pancreatitis. Therefore, in our experience, unnecessary pancreatic resection can be avoided. In addition, no mutation of *KRAS* is found in other pancreatic tumors and cholangiocarcinoma (*i.e.*, different from PDAC); the latter result reinforces the specificity of the ≪*KRAS* assay≫ for PDAC [15,28]. Since *KRAS* analysis is now widely available due to its application as a predictive marker for anti-EGFR therapy in colon cancer, this diagnostic tool could also be applied to help the clinician in managing pancreatic masses. We are making extensive efforts to transfer this technique to clinical practice, as a rapid and cheap determination of *KRAS* mutation analysis using a Taqman-based allelic discrimination protocol.

## 4. Other Molecular Markers for Diagnosis, Prognosis and Treatment for Pancreatic Adenocarcinoma

Large scale analysis of gene expression has been widely proposed as a powerful method for malignancy diagnosis, predicting invasion and metastasis through the identification of biomarkers. Indeed, PDAC has previously been the focus of such studies, including our own experience [29-32]. An important issue is the limited access to pancreatic tissue specimens and validation of some of these markers must be performed on EUS-FNA materials in order to support the clinical relevance of these studies. We (and others) conducted experiments to determine whether quantification of these markers is feasible in EUS-FNA specimens for prognosis or molecular diagnosis. We found that RT-PCR analysis of EUS-FNA samples validated the over-expression of *PLAT* and *LCN2* found in resected tissues following macroarray analysis [32]. Thus, the quality and the amount of cellular sampling using pancreatic EUS-guided FNA allow the extraction of sufficient quantities of RNA to perform RT-QPCR analysis as a new tool for early diagnosis, as described for lymph node metastasis [33]. Furthermore, several authors suggested that FNA could produce a relative enrichment of cancer cells. This enrichment has been attributed to the enhanced aptitude of epithelial cancer cells to be aspirated as compared to stromal cells [34]. These results encourage the use of EUS-FNA along with molecular analysis for the diagnosis and management of pancreatic solid masses. The mRNA levels can also be measured using real-time quantitative RT-PCR formatted in TaqMan low-density array. This *modus operandi* may require RNA amplification. Steg *et al.* used this approach to assay the mRNA levels of the Hedgehog pathway molecules in pancreatic carcinomas samples. They performed EUS-FNA biopsies before and during chemoradiation. While this work demonstrated the feasibility of this technique, no significant changes were observed in the expression of Hedgehog pathway molecules in response to chemoradiation [35]. In the near future, TaqMan low-density arrays could be used to characterize the level of expression of candidate genes implicated in the intracellular metabolism of gemcitabine and, as a consequence, help provide an ≪*a la carte*≫ treatment for epithelial cancers. Recently, it has been demonstrated that patients treated with gemcitabine-based adjuvant chemotherapy, the tumor expression levels of molecules that regulate gemcitabine intracellular metabolism (*hENT1/human equilibrative nucleoside transporter 1; dck/deoxycitidine kinase; CDA/cytidine deaminase; RRM1 and RRM2/ribonucleotide reductases subunits 1 and 2)* could predict the gemcitabine sensitivity (36). This strategy could also be applied in patients for whom gemcitabine-based palliative chemotherapy is proposed, by measuring the expression level of these molecules within the tissue material obtained by EUS-FNAB before treatment.

Several teams succeeded in DNA analysis on EUS-FNA samples to investigate EGFR kinase mutations, EGFR gene amplification, as well as p16, p53 mutations of allelic losses at 19p (bearing p16 tumor suppressor gene) or 18q (bearing DPC4 tumor suppressor gene) chromosomes [37,38]. These monocenter studies evaluated the value of these markers for assessing diagnosis and prognosis of PDAC. Lee *et al.* demonstrated that mutational status of a given gene is feasible in EUS-FNA samples; however they failed to associate the presence of somatic mutations of EGFR, or elevated copy numbers of EGR gene with the prognosis of PDAC [39]. In addition, LOH analysis is also reliable and could be applied for the diagnosis and probably for the prognosis of pancreatic malignancies [27,38]. Along these lines, comparative genomic hybridization (CGH) technology can also be applied to cells obtained by EUS-FNA to assess losses or gains of loci involved in pancreatic carcinogenesis [37]. We and others recently succeeded in detecting and analyzing the expression of microRNA (miRNA) in EUS-FNA samples [40,41]. Safranska *et al.* reported that miRNA levels are affected in PDAC FNAs [42]. The combination of miR-196a and miR-217 segregated PDAC FNA samples from other FNA samples. Also, we recently demonstrated that *let-7* expression is repressed in PDAC FNAs [40]. The level of DNA methylation can be measured by bisulfite mapping and semi-quantitative methylation-specific PCR on EUS-FNA samples. We recently focused our effort on miRNA 148a (miR-148a). We found that its production is repressed, not only in PDAC samples but also during pancreatic carcinogenesis. More importantly, we found that the hypermethylation of the DNA region encoding miR-148a is responsible for its repression. Finally, we showed that the hypermethylated DNA region encoding miR-148a can serve as a new ancillary marker for the differential diagnosis of PDAC [41].

## 5. Conclusions

**Figure 1.**
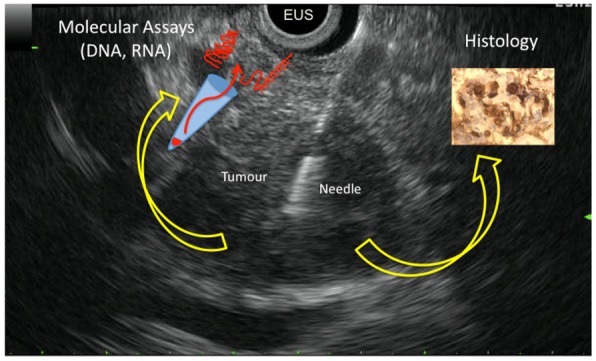
Endoscopic ultrasound picture of a pancreatic solid tumor: the pancreatic tumor appears as a hypoechoic round lesion; the needle biopsy is clearly seen within the tumors (EUS = ultrasonic probe). The scheme illustrates all analyses offered by fine needle biopsy: histology on microbiopsy, DNA and/or RNA extractions on cellular sample obtained after air blushing or rinsing needle after core biopsy reclaim.

EUS-FNAB is a safe and effective technique for diagnosing PDAC, but its accuracy varies widely. However sampling cytological and histological materials is achieved in 85% of patients with PDAC and permits cytopathological diagnosis. The improvement of molecular biology including DNA and RNA amplification enables the analysis and the quantification of candidate molecular markers in cytological samples collected from EUS-FNAB. Analysis can be performed on cellular material sampled by air blushing or rinsing needle once core biopsy is reclaimed. In this way, the results of molecular analysis can be correlated to histology done on the tissue materials collected during the same needle pass. Thus, molecular analysis of EUS-FNAB material is a complementary tool for the diagnosis of PDAC (Figure 1 shows all possibilities of EUS-FNAB). For this indication, *KRAS* mutation analysis is highly promising and ready for use at a clinical level. Other molecular markers (including miRNAs) are yet to be characterized. Moreover, EUS-FNAB allows sampling of biological material from patients with locally advanced PDAC that, unfortunately, represent the vast majority of patients diagnosed with this cancer in clinical practice. In this way, cancer scientists have a unique access to tumor material to perform molecular investigations to better understand the physiopathology, the carcinogenesis, and the response to treatment of advanced PDAC.

## Figures and Tables

**Table 1.** Performances of KRAS mutation analysis alone or associated with cytopathology in materials from EUS-guided FNA for the diagnosis of pancreatic cancer and chronic pancreatitis.

**Author Year [Ref.]**	**Methods for *KRAS* analysis**	**Patients PC/CP**	**Sensitivity (%)**	**Specificity (%)**	**Overall accuracy (%)**
Tada 2002 [22]	PCR + ELMA	28/8	*KRAS: 77* *CytoP: 62* *CytoP* + *KRAS: 81*	*KRAS: 100* *CytoP: 100* *CytoP* + *KRAS:100*	*KRAS: 82* *CytoP: 71* *CytoP* + *KRAS: 85*
Pellisé 2003 [23]	RFLP	33/24	*KRAS: 73* *CytoP: 97* *CytoP* + *KRAS: 97*	*KRAS: 100* *CytoP: 100* *CytoP* + *KRAS: 100*	*KRAS: 91* *CytoP: 84* *CytoP* + *KRAS: 98*
Takahashi 2005 [23]	PCR-SSCP	62/15	*KRAS: 74* *CytoP: 84* *CytoP* + *KRAS: 94*	*KRAS: 100* *CytoP: 100* *CytoP* + *KRAS: 100*	*-* *CytoP: 58* *-*
Maluf-Filho 2007 [25]	RFLP	57/11	*CytoP: 82* *CytoP* + *KRAS: 90*	*CytoP: 97* *CytoP* + *KRAS: 47*	*CytoP: 59* *CytoP* + *KRAS: 89*
Salek 2007 [26]	CGCE	81/20	*KRAS: 70*	*KRAS: 100*	*-*
Bournet 2009 [15]	RFLP + sequencing	129/27	*KRAS: 67* *CytoP: 83* *CytoP* + *KRAS: 88*	*KRAS: 100* *CytoP: 100* *CytoP* + *KRAS: 100*	*KRAS: 86* *CytoP: 72* *CytoP* + *KRAS: 90*

PC: pancreatic cancer; CP: chronic pancreatitis; CytoP: cytopathology; PCR: polymerase chain reaction; EMLA: Enzyme linked mini-sequence assay; PCR-SSCP: PCR-single strand conformation polymorphism; RFLP: restriction fragment length polymorphism; CGCE: cycling-gradient capillary electrophoresis.
